# Peripheral surrogates of tumor burden to guide chemotherapeutic and immunotherapeutic strategies for HPV-associated malignancies

**DOI:** 10.18632/oncotarget.28487

**Published:** 2023-08-10

**Authors:** Meghali Goswami, Jeffrey Schlom, Renee N. Donahue

**Affiliations:** ^1^Center for Immuno-Oncology, National Cancer Institute, National Institutes of Health, Bethesda, MD 20892, USA

**Keywords:** HPV-associated malignancies, immunotherapy, circulating tumor DNA, circulating tumor cells, HPV-specific antibodies

## Abstract

With the rapid adoption of immunotherapy into clinical practice for HPV-associated malignancies, assessing tumor burden using “liquid biopsies” would further our understanding of clinical outcomes mediated by immunotherapy and allow for tailoring of treatment based on real-time tumor dynamics. In this review, we examine translational studies on peripheral surrogates of tumor burden derived from peripheral blood in HPV-associated malignancies, including levels and methylation of circulating tumor DNA (ctDNA), miRNA derived from extracellular vesicles, circulating tumor cells (CTCs), and HPV-specific antibodies and T cell responses. We review their utility as prognostic and predictive biomarkers of response to chemotherapy and radiation, with a focus on how they may inform and guide immunotherapies to treat locally advanced and metastatic HPV-associated malignancies. We also highlight unanswered questions that must be addressed to translate and integrate these peripheral tumor biomarkers into the clinic.

## INTRODUCTION

Persistent infection with high-risk human papillomavirus (HPV) subtypes can pave the way for oncogenic transformation and development of HPV-driven malignancies. Globally more than 600,000 cases of HPV-associated cancers occur annually, representing about 5% of all cancers. Estimates of five-year overall survival (OS) vary between 37% to 62%. HPVs are small, non-enveloped double-stranded viruses that infect the basal layer of stratified squamous epithelium, and the most common sites of infection include the head and neck, cervix, and anus. Thus, oropharyngeal, cervical, and anal cancers are more prevalent, but rarer vaginal and penile cancers also occur [1].

The HPV viral genome is comprised of early (E), late (L), and long control (LCR) proteins. The early proteins E6 and E7 are main drivers of malignant transformation, as they interfere with p53 and retinoblastoma (RB) tumor suppressor pathways, respectively. Also often accompanying HPV-driven tumorigenesis are mutations in key cell signaling genes (*PIK3CA*, *PTEN*, genes encoding antigen-presenting cell (APC) machinery, among others) and alterations in epithelial-like and mesenchymal cell states [2, 3]. HPV can also dampen interferon (IFN) responses and antigen presentation; HPV alterations to host immunity have been previously comprehensively reviewed [1, 4]. Current immunotherapies under active investigation in HPV-associated malignancies include immune checkpoint inhibition (ICI), vaccines, immunocytokines, adoptive cell therapies, PD-L1/TGFβ1 inhibition, and combinations of these modalities, as the expression of viral E6 and E7 oncogenic proteins makes these cancers amenable to such immunotherapeutic strategies [5–7]. Approximately 20% of HPV-associated malignancies may derive benefit from PD-(L)1 ICI, but a substantial number of patients will eventually develop ICI-refractory disease [8, 9]. For most of these patients, there is no current effective standard of care therapy.

Monitoring clinical progress in patients treated with traditional chemotherapy and radiation therapy (CRT) and immunotherapies is typically measured using RECIST version 1.1 criteria [10]. Evidence, primarily derived from the HPV+ head and neck squamous cell carcinoma (HNSCC) setting, indicates considerable tumor inter- and intra-patient heterogeneity, as the driving HPV subtype and accompanying genetic alterations lead to multiple genomic subtypes of disease [11]. Intratumoral heterogeneity in HPV+ oropharyngeal squamous cell carcinoma (OPSCC) tissue has been shown to be associated with worse survival after CRT and has prognostic value in this setting [12, 13]. However, directly interrogating the tumor for evaluation of tumor phenotype and/or the immune microenvironment is exceptionally difficult, as obtaining primary and secondary lesions for examination is invasive, painful and not feasible at frequent intervals. Sample quality and timing issues are also relevant. However, tumor and/or immune signatures derived from peripheral blood are minimally invasive and allow for more frequent assessment and surveillance of tumor burden, anti-tumor activity, and patient response to treatment [14], and could be used to complement methods that directly interrogate the tumor microenvironment.

**Figure 1:**
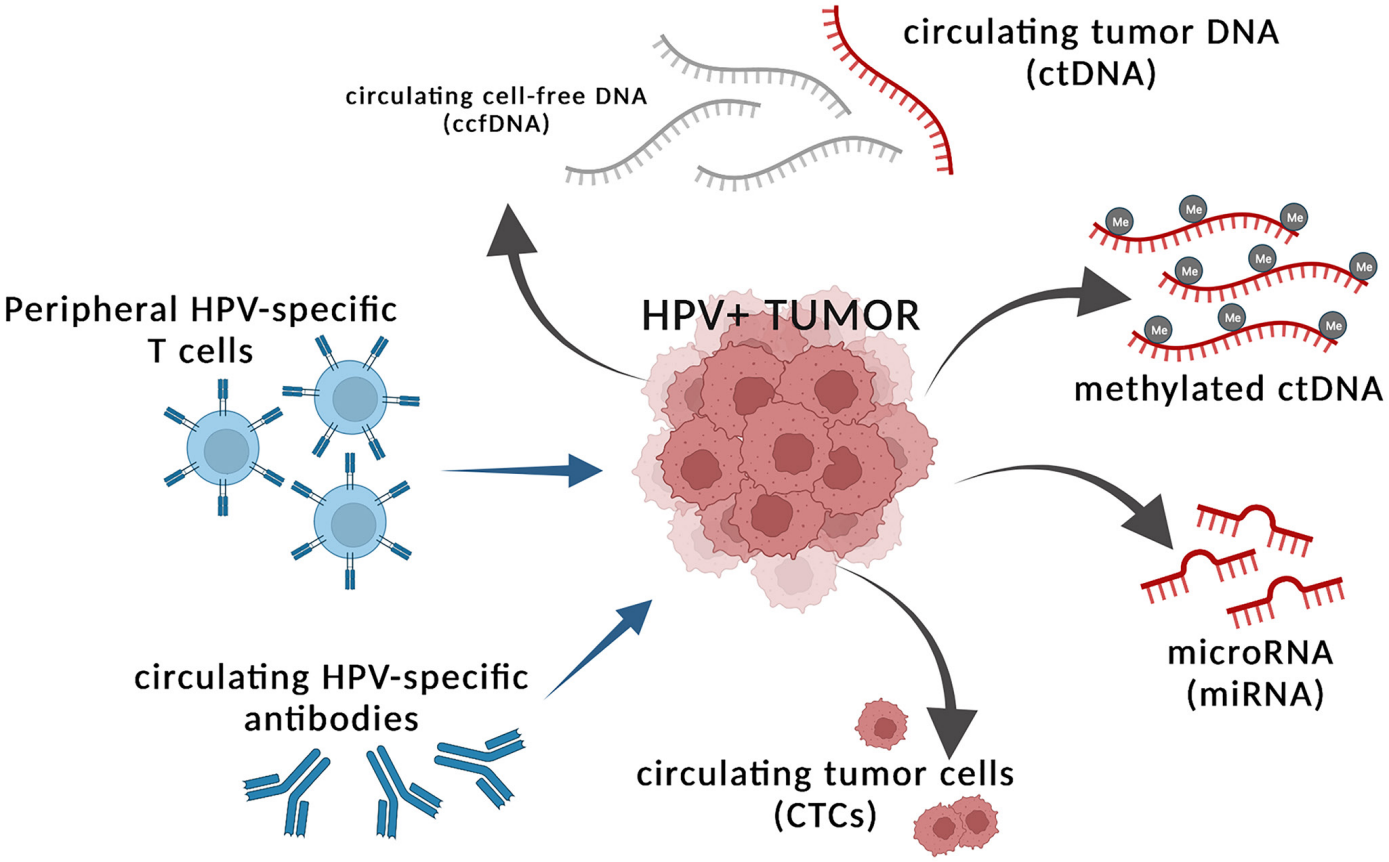
Tumor intrinsic and extrinsic factors in the periphery that can serve as proxies of tumor burden. HPV+ tumors shed circulating tumor DNA (ctDNA) that can be detected within the greater circulating cell-free DNA (ccfDNA) fraction and indicate presence of tumor. The ctDNA may harbor differential methylation patterns, which can be used as tumor intrinsic peripheral surrogates of tumor burden. HPV+ tumors also shed microRNAs (miRNA) and circulating tumor cells (CTCs) into the periphery, which can reflect tumor burden. Tumor extrinsic factors that may reflect presence of tumor include changing levels of peripheral HPV-specific T cells and HPV-specific antibodies, potentially indicating ongoing immune activity against cancerous cells. Figure created with https://www.biorender.com/.

We believe that peripheral surrogates of tumor burden can serve as dynamic and informative companions to disease monitoring for comprehensive assessment of immunotherapeutic strategies. Here, we review several proxies of tumor burden in the periphery of patients with HPV-associated cancers, including circulating tumor DNA (ctDNA), non-coding RNA, circulating tumor cells (CTCs), and HPV-specific antibodies and T cells (Figure 1). We discuss existing clinical data on these surrogates of tumor burden and their potential in evaluating efficacy of immunotherapy in HPV-associated malignancies.

## CIRCULATING CELL-FREE NUCLEIC ACIDS

### Circulating tumor DNA (ctDNA)

Apoptosis, necrosis, and other cellular turnover processes in both healthy individuals and in those with cancer lead to the release of fragmented circulating cell-free DNA (ccfDNA) into the circulation, typically less than 200 base pairs (bp) in length [15]. These ccfDNA fragments are detectable in human plasma and serum, and in patients with cancer, some fraction of this ccfDNA reflects circulating tumor DNA (ctDNA). In HPV-associated malignancies, efforts to analyze ctDNA predominantly focus on detection of E6 and/or E7 regions of high-risk HPV subtypes, as these oncogenic sequences are reflective of HPV integration, and not normal physiological cell death.

Presently, measuring circulating tumor HPV DNA (ctHPV-DNA) is not widely used as a clinically ready diagnostic for HPV-associated malignancies, though several studies have gauged concordance between ctHPV-DNA measurement and traditional clinical tests using primary tumor tissue to discern HPV positivity. These diagnostics include immunohistochemistry of p16 protein, *in situ* hybridization of E6/E7 transcripts, and HPV PCR of primary tumor [16]. By far, most data exist for HNSCC, and particularly for OPSCC.

Recently, studies have embraced the use of digital droplet polymerase chain reaction (ddPCR) to capture ctHPV-DNA in HPV-associated malignancies, as the assay is a highly specific and sensitive method for absolute quantification. Reports evaluating ctHPV-DNA for HNSCC/OPSCC using ddPCR targeting E6 and/or E7 genes of HPV-16, HPV-18, and/or other high-risk HPV subtypes show a composite concordance of about 88% (with ranges from 56% to 100% across studies) with clinically determined HPV genotype, without appreciable differences between newly diagnosed, locally advanced, or metastatic disease states [17–27]. The sensitivity of ddPCR based assays to detect ctHPV-DNA appears lower in HPV-positive cervical cancers, as studies in this setting report concordances of 62–69% for locally advanced disease [28–30]. However, in a small cohort of patients with metastatic cervical cancer, the sensitivity of the ddPCR assay was 100% [31]. Sensitivity estimates of ddPCR assays in both locally advanced and metastatic anal cancers range from 88–93% [27, 32, 33], but to date, data on ctHPV-DNA positivity in rarer HPV-associated malignancies have not been reported. Table 1 details sensitivity and specificity measures from three reports where study design enabled these calculations.

**Table 1:** Performance characteristics of ctHPV-DNA assessment in distinguishing between HPV+ and HPV− cases

Patient cohort	Assay	ctHPV-DNA result	HPV+ (*n*)	HPV− (*n*)	Sensitivity	Specificity	PPV	NPV	Reference
OPSCC	NavDx	ctHPV-DNA+	92	3	89%	97%	97%	91%	[22]
		ctHPV-DNA−	11	112					
cervical, anal, OPSCC	HPV16_E7 and HPV18_E7 ddPCR	ctHPV-DNA+	61	0	87%	100%	100%	67%	[27]
		ctHPV-DNA−	9	18					
cervical	HPV16_E7 and HPV18_E7 ddPCR	ctHPV-DNA+	19	0	100%	100%	100%	100%	[31]
		ctHPV-DNA−	0	45					

HPV+ and HPV− determined by clinical diagnostic. HPV− cohorts include healthy donors, cervical intraepithelial neoplasms, and confirmed HPV-negative malignancies. Abbreviations: PPV: positive predictive value; NPV: negative predictive value.

A complicating factor is the lack of a consistent ddPCR assay for the detection of ctHPV-DNA across studies, as some groups have evaluated E6 and/or E7 for the most common high-risk HPV subtypes (HPV 16 and 18), whereas other groups have measured E6 and/or E7 from additional high-risk subtypes (HPV 31, 33, 35). In a cohort of locally advanced cervical cancer, several additional ddPCR assays were required to capture all patients (HPV 45, 52, 73) [30]. The NavDx platform, based on the work of Chera, et al. employs an iterative approach by first measuring HPV-16 and only measuring other high-risk HPV subtypes if HPV-16 is negative [22, 34].

### Kinetics of ctHPV-DNA during CRT

Most of the existing evidence evaluating the utility of ctDNA as a biomarker for clinical response to treatment is derived from the setting of chemotherapy and radiation (CRT). In several studies of ctHPV-DNA in the CRT setting, there has been no strong association between baseline ctHPV-DNA burden and clinical outcome, as studies in HNSCC, cervical, and anal cancers have not found baseline ctHPV-DNA to be associated with progression-free survival (PFS) or overall survival (OS) [29, 32, 33]. This indicates that the kinetics of changes in ctHPV-DNA during therapy may have more prognostic value than baseline ctHPV-DNA.

Early changes and serial sampling post CRT-initiation may have prognostic value and identify patients more likely to respond to treatment. In a cohort of 67 patients with HPV+ OPSCC with available weekly peripheral bloods during treatment, a subset of 19 patients with baseline ctHPV-DNA load of >200 copies/ml had >95% clearance of ctHPV-DNA by 4 weeks after treatment, and all achieved complete response (CR) to CRT [22]. Conversely, in 28 patients with locally advanced HPV+ OPSCC with detectable baseline ctHPV-DNA, changes in ctHPV-DNA at week 4 compared to baseline were not associated with outcome; however, an early increase in ctHPV-DNA at week 2 was in fact associated with less tumor progression [26]. Another study of locally advanced cervical cancer showed a transient increase in ctHPV-DNA in 6 of 14 evaluable patients between 1–3 weeks after start of CRT, where ultimately 3 patients relapsed while 3 did not [30]. While seemingly counterintuitive, these reports suggest that very early increases in ctHPV-DNA may reflect tumor cell death and release of tumor fragments into the circulation, and not necessarily tumor progression.

**Figure 2:**
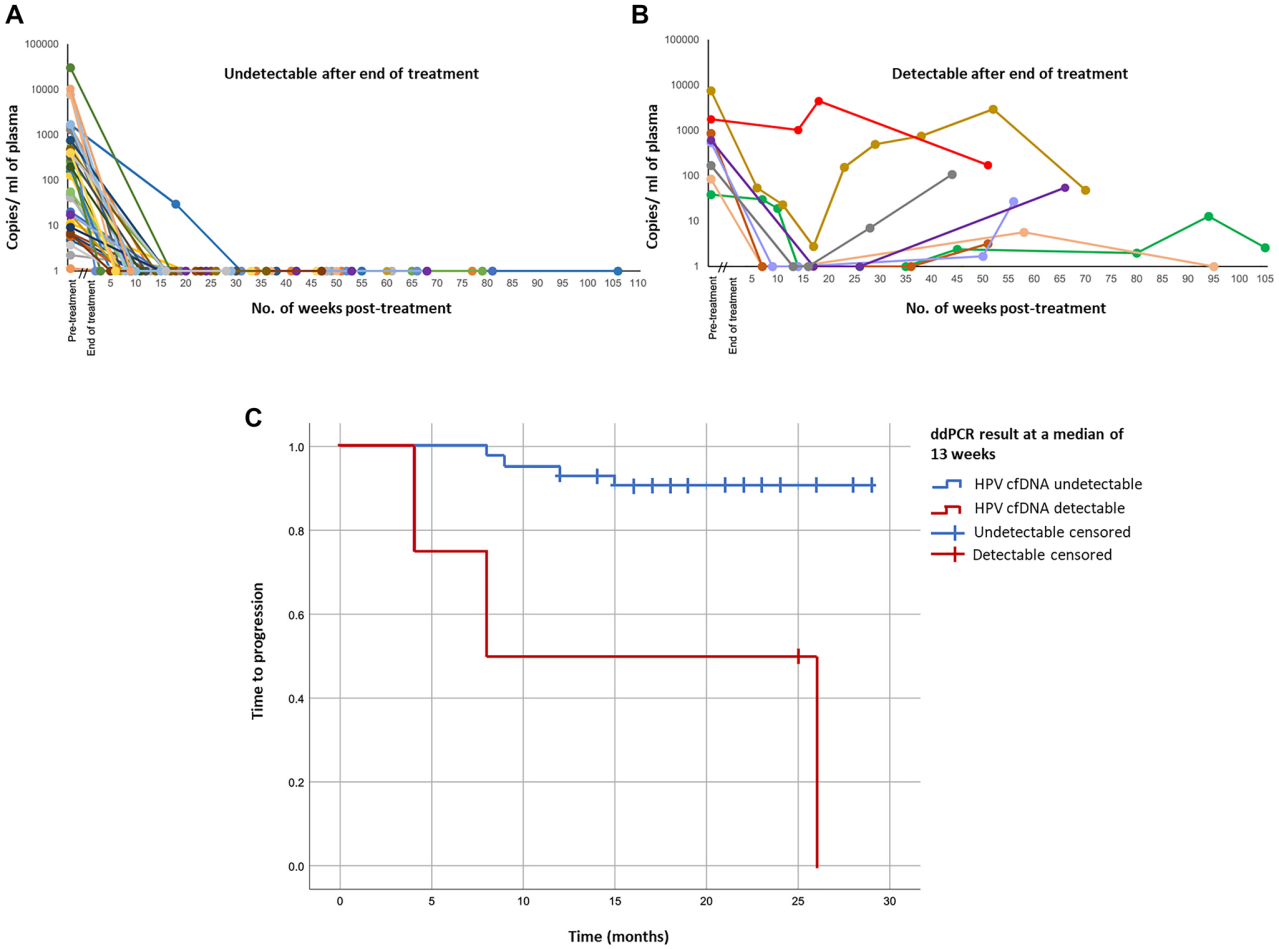
Treatment responses and patient outcomes in HPV-positive patients according to plasma ctHPV-DNA copy number. (**A**) ctHPV-DNA copy number in patients for whom ctHPV-DNA levels became undetectable following treatment and remained undetectable for duration of follow-up (*n* = 40). (**B**) ctHPV-DNA copy number in patients whose ctHPV-DNA levels never became undetectable following treatment or became undetectable but subsequently increased (*n* = 8). (**C**) Kaplan-Meier plot of time to progression for all patients with detectable ctHPV-DNA pre-treatment with subsequent post-treatment samples (*n* = 48), stratified by presence or absence of detectable ctHPV-DNA at a median of 13 weeks post-treatment. Figure adapted from Warlow SJ, Eur J Surg Oncol 2022 [24]. Copyright © Warlow et al. Published by Elsevier Ltd.

Levels of and changes in ctHPV-DNA during later phases of CRT treatment have also shown to be associated with disease recurrence. In a cohort of 47 patients with any-stage OPSCC during treatment with CRT and/or surgery, 40 of these patients had undetectable ctHPV-DNA 2–22 weeks into treatment and remained disease-free for the duration of follow-up, while remaining patients had detectable ctHPV-DNA as high as 10,000 copies/ml (Figure 2A, 2B). Detection of ctHPV-DNA around the time of 12-week restaging scans effectively stratified patients by 1 year time to progression (TTP), with 50% of patients with undetectable ctHPV-DNA versus 93% of those who had detectable ctHPV-DNA after the start of CRT progressing [24] (Figure 2C).

Detection of ctHPV-DNA soon after the completion of CRT to several months post-treatment is strongly associated with clinical outcomes. In a cohort of HPV16+ advanced anal cancer, 14 of 36 evaluable patients who had detectable ctHPV-DNA after completion of the CRT course had significantly worse PFS than 22 patients without residual ctHPV-DNA (3.4 months vs. not reached, respectively) [33]. Another study in 33 locally advanced anal cancer reported similar findings, as detectable ctHPV-DNA after CRT was strongly associated with disease recurrence [32]. Similarly, in a study of 94 patients with HPV16+ or HPV18+ cervical cancer, complete clearance of ctHPV-DNA by the end of CRT was associated with longer PFS [29]. Importantly, 16 of these patients eventually developed relapsed disease during follow-up post-CRT, and detection of ctHPV-DNA preceded clinical diagnosis of relapse by 2–15 months. However, a caveat emerges from a study of 115 non-metastatic HPV+ OPSCC patients. In post-CRT surveillance, 24 patients had an increase in ctHPV-DNA at some point during follow-up; 15 of these patients did develop disease recurrence and had consistently elevated ctHPV-DNA, while 8 of these patients had a transient elevation in ctHPV-DNA that became undetectable at the next available timepoint [34]. This highlights the importance of serial sampling in the surveillance period to confirm a positive ctHPV-DNA result, and it also suggests the possibility of a role for immune-mediated control and eradication of local disease recurrence. Importantly, the ability to detect ctHPV-DNA much earlier than overt clinical disease suggests another possible use of ctHPV-DNA in a post-exposure surveillance setting, where routine monitoring of individuals at high risk for developing HPV-associated malignancies could enable early disease detection.

Several clinical trials in the United States have begun integrating the measurement of ctHPV-DNA in both retrospective and prospective manners in patients with HPV+ cancers, all in the CRT setting (Table 2). The data generated from these studies will hopefully lead to the incorporation of serial ctHPV-DNA monitoring into clinical response assessment and treatment strategies in the CRT and eventually immunotherapy settings.

**Table 2:** Clinical trials incorporating ctHPV-DNA assessment as of April 19, 2023 in the United States

Identifier	Clinical trial name	Patient cohort	Treatment agents
NCT04564989	Prospective Observational Study to Validate Circulating HPVDNA and Prognostic Genomic Biomarkers in HPV-associated OPSCC	HPV+ OPSCC	Curative-intent treatment
NCT05541016	Blood-Based Biomarkers to Inform Treatment and Radiation Therapy Decisions for HPV Associated Oropharyngeal Squamous Cell Head and Neck Cancers - DART 2.0	HPV+ OPSCC	Standard of care surgery; diffusing alpha-emitter radiation therapy + docetaxel; intensity-modulated radiation therapy +/− cisplatin
NCT04900623	Risk-adapted Therapy in HPV+ Oropharyngeal Cancer Using Circulating Tumor (ct)HPV DNA Profile - The ReACT Study	HPV+ OPSCC	Radiation therapy; cisplatin, carboplatin, or paclitaxel
NCT04965792	Post-treatment Surveillance in HPV+ Oropharyngeal SCC	HPV+ OPSCC	Curative-intent treatment
NCT05606133	Circulating Human Papilloma Virus (HPV) DNA for the Screening and Surveillance of Gynecologic Cancers	HPV+ cervical dysplasia and cancer	Curative-intent treatment
NCT04857528	Detecting HPV DNA in Anal and Cervical Cancers	HPV+ cervical and anal cancers	Radiation therapy
NCT05307939	A Study on Using Cell-Free Tumor DNA (ctDNA) Testing to Decide When to Start Routine Treatment in People With Human Papilloma Virus (HPV)-Associated Oropharynx Cancer (OPC)	HPV+ OPSCC	Surveillance; adjuvant radiation therapy; cisplatin or carboplatin

### Kinetics of ctHPV-DNA during immunotherapy in HPV-associated cancers

To date, surprisingly little evidence has been documented on ctHPV-DNA kinetics and relationships to clinical outcome in patients with HPV-associated malignancies receiving various modalities of immunotherapy. Existing data come from studies in small cohorts of metastatic HNSCC and cervical cancers.

**Figure 3:**
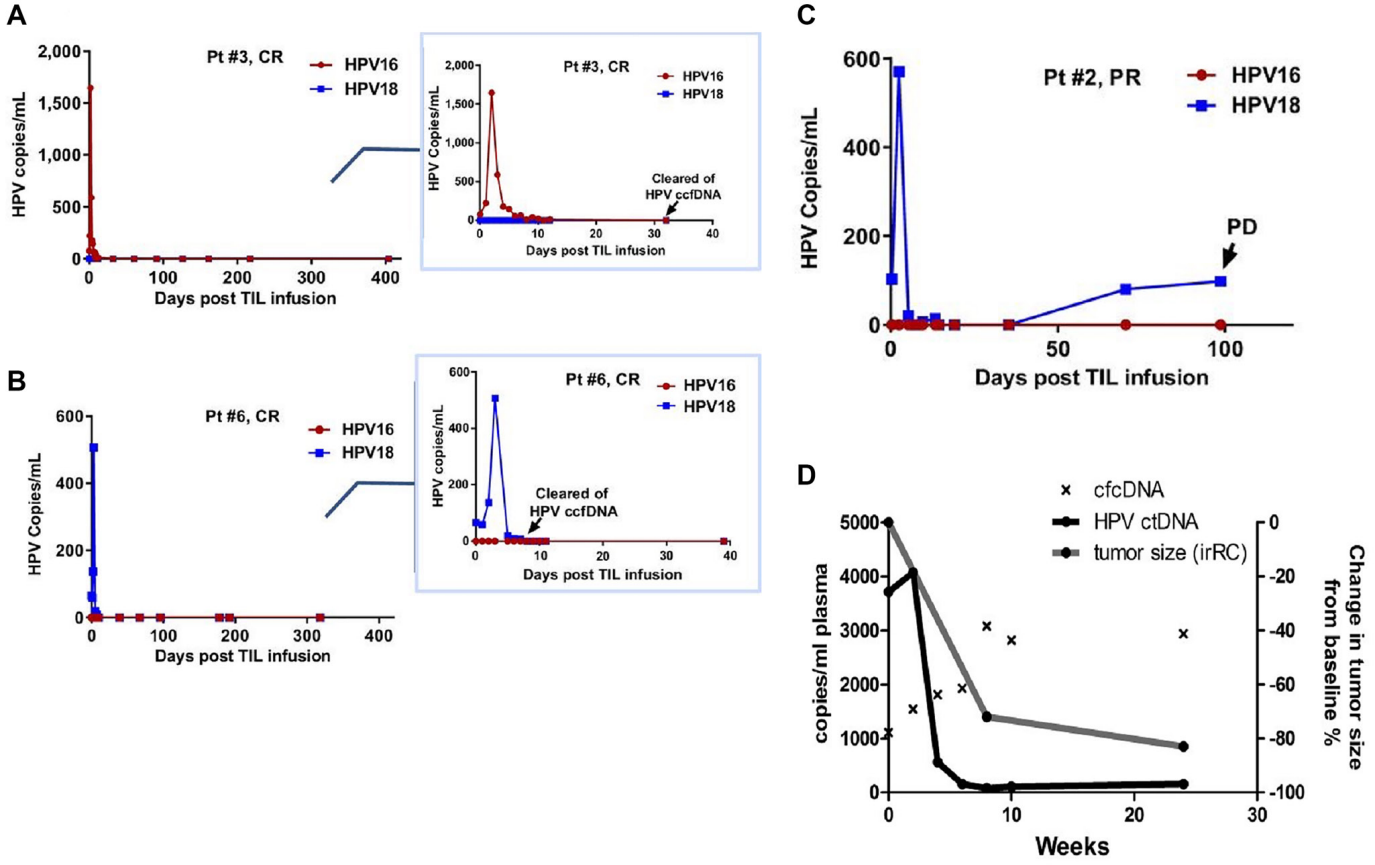
Early transient increases in ctHPV-DNA in patients with HPV-associated malignancies treated with immunotherapies. (**A**–**C**) HPV copies per ml of serum in patients with metastatic cervical cancer treated with experimental TIL immunotherapy. Inserts zoom in on the first 40 days post-TIL infusion. Abbreviations: CR: complete response; PR: partial response; PD: progressive disease. (**D**) HPV copies per ml of plasma in a patient with metastatic anal SCC treated with nivolumab. cfcDNA is measure of copies of RPP30 control gene per ml of plasma. Figures from Kang Z, Clin Cancer Res 2017 [31]. Copyright © 2017, American Association for Cancer Research; and Cabel L, Int J Cancer 2017 [36]. Copyright © John Wiley & Sons, Inc.

Kang, et al. reported ctHPV-DNA kinetics in 9 patients with HPV+ metastatic cervical cancer receiving a single infusion of tumor-infiltrating lymphocytes (TILs) reactive against HPV E6 and E7 [35]. Of these patients, 6 had consistent progressive disease (PD) and remained positive for ctHPV-DNA at all timepoints evaluated, whereas the 3 patients who responded to therapy (complete or partial responses, CR or PR, respectively) turned negative for ctHPV-DNA soon after treatment initiation [31]. Curiously, the 3 patients who achieved CR/PR experienced a very transient but striking and immediate increase in ctHPV-DNA 2–3 days after TIL infusion that quickly reverted to low/undetectable levels (Figure 3A–3C). Another group reported as a case report ctHPV-DNA dynamics in a patient with metastatic anal SCC receiving nivolumab also at a q2w schedule [36]. Here, a transient 10% increase in ctHPV-DNA immediately after the second dose of nivolumab was documented, after which ctHPV-DNA dropped by over 95% from baseline after 4 doses (Figure 3D). It is notable that this brief increase in ctHPV-DNA immediately after treatment initiation was both observed in patients receiving TIL therapy as well as in a patient receiving nivolumab all of whom achieved clinical responses. In the patient with metastatic SCC treated with nivolumab, at 24 weeks there was an 80% reduction in tumor size from baseline, and ctHPV-DNA remained detectable through 24 weeks of follow-up, at low levels of around 100 copies/ml of plasma [36]. This suggests ongoing immune control of residual tumor; however, it also raises the concern for immuno-editing of the tumor and eventual disease progression.

**Figure 4:**
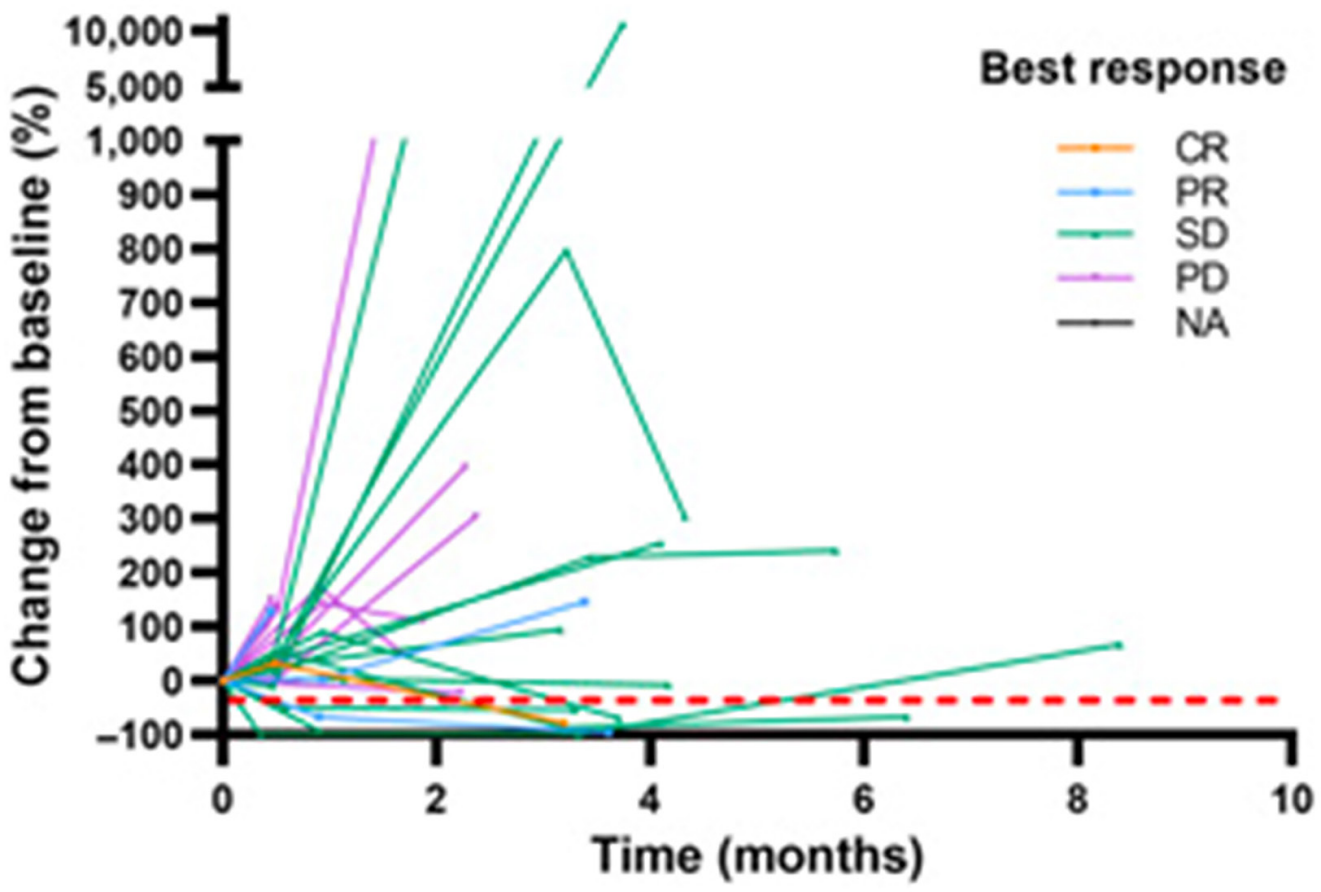
Longitudinal ctHPV-DNA levels in patients with HPV-associated malignancies treated with cetuximab and nivolumab. Spider plot to illustrate percent changes in TTMV DNA levels in 34 pl6+ patients with available baseline TTMV-DNA. The best response was determined by RECIST criteria. Abbreviation: NA: not applicable. Figure from Chung CH, Clin Cancer Res [17]. Copyright © 2022, American Association for Cancer Research.

In a cohort of 37 HNSCC patients with recurrent and/or metastatic disease receiving the combination of the epidermal growth factor inhibitor cetuximab and nivolumab on a q2w cycle for up to 24 cycles with evaluable plasma samples, ctHPV-DNA was measured with the NavDx assay, where results are reported as composite TTMV-DNA copies/ml, encompassing multiple high-risk HPV subtypes. In this study, response rate, defined as CR/PR, was higher in patients who had <1230 copies/ml of TTMV-DNA at baseline. These patients also had longer median PFS (8 vs. 3 months) and longer OS (20 vs. 9 months) than patients with >1230 copies/ml of TTMV-DNA respectively at baseline [17]. Of note, some patients with PD on this trial had roughly a 10000% increase from baseline in TTMV-DNA during treatment with cetuximab and nivolumab (Figure 4).

**Figure 5:**
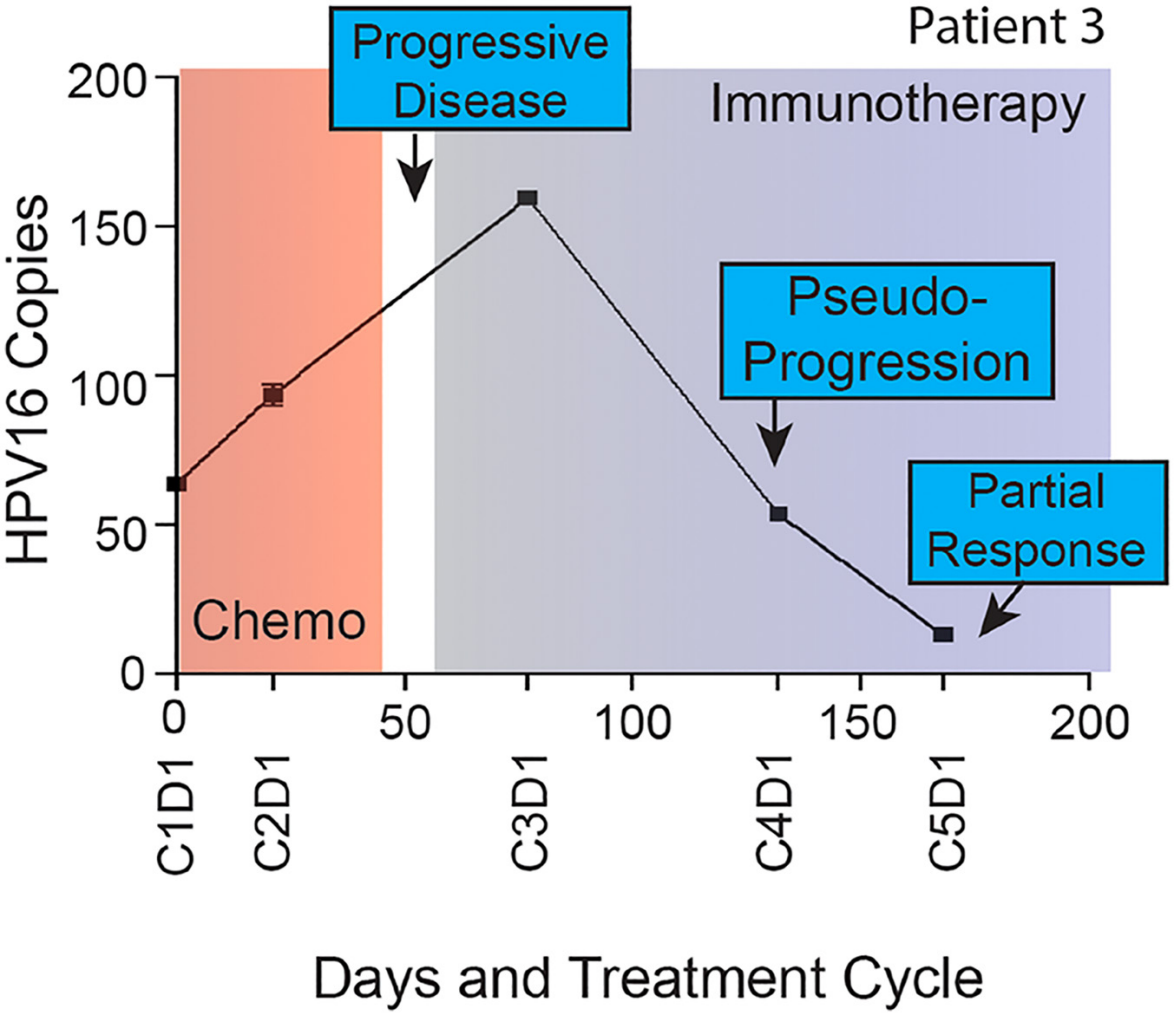
Longitudinal ctHPV-DNA levels in a patient with HPV-16+ malignancy treated with unspecified immunotherapy. Plasma HPV16 ctDNA (copies per l uL of plasma) measured over time in a patient with p16+ recurrent/metastatic HPV+ OPSCC. On the x-axis are days since study enrollment as well as cycle and day (CxDx) of treatment in a patient with radiographic pseudo-progression while treated with immunotherapy. Figure from Haring CT, Oncotarget 2021 [23]. Copyright © Haring et al.

In a study of recurrent and/or metastatic HNSCC receiving chemotherapies (*n* = 11) and unspecified immunotherapies (*n* = 7), up to a 60% increase or any decrease in ctHPV-DNA levels during treatment was associated with response (here defined as CR, PR, or stable disease (SD)), while a greater than 60% increase after 1 cycle of treatment was associated with PD [23]. In another cohort of 22 patients with advanced HPV+ OPSCC where patients received chemotherapy or unspecified immunotherapy, the rate of change in levels of ctHPV-DNA differed between patients receiving immunotherapy versus cytotoxic chemotherapy (median decrease in ctHPV-DNA of 3.6% vs. 5.3% per day, respectively) [37]. Across these studies, patients with PD had increases in ctHPV-DNA that always preceded restaging scans. Of particular interest, radiographic pseudo-progression was documented in 2 patients receiving immunotherapy and first scans suggested PD, though ctHPV-DNA decreased from baseline levels [23]. These patients continued receiving immunotherapeutic treatment and eventually achieved a clinical response, pointing towards a role for ctHPV-DNA in resolving pseudo-progression versus actual tumor growth (Figure 5).

Together, these studies highlight potential clinical validity and utility of longitudinal ctHPV-DNA monitoring during immunotherapeutic treatment of HPV-associated malignancies in observing responses before first restaging scans, predicting clinical response and progressive disease, resolving clinical events like pseudo-progression, and detecting residual disease.

### Sequencing of ctHPV-DNA

The detection of ctHPV-DNA is readily amenable and straightforward with ddPCR; however, the above studies suggest a reliable lower limit of detection (LLOD) of about 1 copy/ml of serum or plasma. Sequencing of circulating cell free DNA (ccfDNA) has the potential for even greater sensitivity of ctHPV-DNA; however, the process of generating sequencing libraries, especially from small fragments of ccfDNA, can be prone to quantitative biases and artifacts introduced by PCR amplification [38]. In one study of patients with locally advanced cervical cancer, the authors developed and evaluated a dual-strand viral genome hybrid capture sequencing method, termed HPV-sequencing, or HPV-seq. The authors had previously reported that the detection of ctHPV-DNA in cervical cancer patients after standard of care CRT was associated with worse PFS and preceded clinical diagnoses of recurrence and/or metastases [39]. HPV-seq performed on these same patients yielded concordant results with ddPCR but also allowed for detection of ctHPV-DNA as low as 0.03 copies/ml of plasma [40]. However, several patients were negative for ctHPV-DNA by ddPCR but positive by HPV-seq but had not experienced disease recurrence for 3 or more years of follow-up in this study. Another study employed targeted sequencing of E6 and E7 regions from 13 high-risk HPV genotypes from plasma in 35 patients with cervical cancer undergoing CRT and/or surgery. Here, 100% of patients with advanced disease had detectable ctHPV-DNA of any genotype, whereas ctHPV-DNA was detected in 56% of patients with early-stage disease. Similar to above, targeted sequencing of E6/E7 led to continued detection of ctHPV-DNA from one patient who achieved and maintained remission for at least 2 years after radiotherapy and bevacizumab [41]. A study using a different sequencing assay of 8 high-risk genotypes (termed panHPV-detect) in HPV+ HNSCC, cervical, and anal cancers reports on one patient who achieved CR after CRT but was positive for ctHPV-DNA by panHPV-detect. This patient ended up with distal relapse 9 months after the completion of CRT. Four patients in this study had abnormal imaging post CRT but undetectable ctHPV-DNA, and all of these patients had not relapsed by 12 months after CRT [42]. These discordances between ctHPV-detection using highly sensitive sequencing methods and disease recurrence warrant more research to understand whether exceedingly low levels of ctHPV-DNA are associated with disease recurrence or reflect presence of past disease.

### ctDNA methylation signatures

There has been recent movement towards evaluating epigenetic signatures including DNA methylation in cancer. Profiling of peripheral blood for tumor-based DNA methylation profiles for the early detection of cancer is an active area of exploration [43]. Recent reviews present comprehensive summaries of the known relationships between ctDNA methylation and clinical outcomes in cervical, breast, prostate, lung, and colorectal cancers [44, 45]. Several studies have examined whether promoter methylation of specific genes can aid in the detection of low to high grade cervical intraepithelial lesions. Plasma methylation of HPV genes, including L1, as well as promoter methylation of *CADM1*, *CDH1* (promoter for E-cadherin), and *CDH13* (cadherin-13), among others, readily distinguishes between cancer and benign/no disease controls [46, 47]. Hypermethylation of *CDH1* and *CDH3* in a cohort of patients with cervical cancer was associated with worse disease-free survival [47]. A study identified a set of genes with differentially methylated promoters that could distinguish HPV+ OPSCC from healthy donors using plasma-derived ctDNA. Furthermore, in this study, patients with tumor regression during CRT had decreasing levels of methylated ctDNA at *CALML5*, *DNAJC5G*, and *LY6D*, while patients with PD maintained stable methylation profiles of these genes, suggesting a possible role for ctDNA methylation as a biomarker of response [48]. To our knowledge, no data exist on ctDNA methylation profiles in HPV-associated malignancies in the setting of immunotherapy, but with advancing research on the prospective use of ctDNA as a biomarker of disease burden, we anticipate future studies evaluating ctDNA methylation status as a predictor of response in this setting. Additionally, evaluation of ctDNA and ctHPV-DNA methylation signatures may yield greater insights into mechanisms of action as HDAC inhibitors, including entinostat, in combination with other immunotherapeutic agents are moving into clinical trials for the treatment of immune checkpoint refractory HPV-associated malignancies and other solid tumors [49, 50].

### miRNA from serum- or plasma-derived extracellular vesicles

MicroRNAs (miRNAs) are non-coding RNAs about 20 nucleotides in length that have been implicated in the development of many human cancers. Dysregulation of miRNAs can lead to alterations in many cellular processes that promote oncogenesis [51]. Tumor-specific miRNAs can be carried away from the primary tumor and into the circulation as cargo of tumor-derived extracellular vesicles (EVs) released into the periphery, which have been shown to have potential as biomarkers in several cancers [52]. Several studies have examined the diagnostic ability of plasma exosomal miRNAs in distinguishing between healthy donors (HD) and patients with cervical intraepithelial neoplasms (CIN) and/or cervical carcinoma. In a cohort of 97 patients with cervical cancer, miR-146a-5p, miR-15a-3p, and miR-2110 were upregulated in patients compared to healthy controls, and the former two miRNAs were also upregulated in tumor specimens, suggesting miRNAs came from the tumor and are a direct reflection of tumor burden [53]. Additional plasma-derived miRNAs, including miR-125a-5p, miR-30d-5p, and let-7d-3p, have discriminated between HD, CIN, and cervical carcinoma, and could in fact distinguish different CIN stages [54, 55]. Curiously, miR-125a-5p was upregulated to a lesser extent in HPV+ cervical cancer patients than those with HPV-negative disease, indicating that miRNA profiles in HPV-driven malignancies may differ from cancers without viral etiology [54]. Serum EV-derived long non-coding RNA (lncRNA) DLX6-AS1 was significantly higher in 114 patients with cervical cancers compared to patients with CIN and HD, and its detection after tumor resection was associated with relapse, metastases, and overall worse clinical outcomes [56].

Similar results have been reported in HPV+ HNSCC. In a cohort of patients with locally advanced p16+ OPSCC, a panel of miRNAs was assembled that accurately distinguished OPSCC from healthy donors with 90% sensitivity [57]. In another study of locally advanced HNSCCs, after interrogation of discovery and validation cohorts, 4 miRNAs were able to discriminate well between HNSCC and HD. In particular, miR-491-5p correlated with clinical stage, and changes in the expression of this miRNA within EVs was associated with 1-year disease recurrence after CRT [58]. While data on changes in miRNA expression during treatment with either CRT or immunotherapy are not available, these studies suggest the potential of serum-derived miRNA signatures in HPV-driven disease detection and possibly predicting patient outcome. The serum- and plasma-derived miRNAs and lncRNAs highlighted here may have increased utility together as a panel in the detection and monitoring of HPV-associated cancers in the CRT and immunotherapy settings, and more in-depth studies in these areas are warranted.

## CIRCULATING TUMOR CELLS (CTCs)

Circulating tumor cells (CTCs) can disseminate from the primary tumor into the periphery and have the propensity to seed metastases at distant sites [59, 60]. These cells can be detected as a single cell or as CTC aggregates in peripheral blood, and platforms can distinguish CTCs based on size or expression of select markers. However, CTC capture efficiency is low and more comparative research between methods of CTC enrichment are needed [61].

Limited data exist on CTCs in HPV-associated malignancies, with most data coming from studies performed in HPV+ HNSCC. In one study, CTCs were enriched from blood from patients with HNSCC using the ClearCell FX system, which separates cells based on size. CTCs, both as single cells and in clusters, were detected in 10/23 (43%) patients, and these patients also had shorter PFS after CRT than patients without detected CTCs [62]. Another group enriched for CTCs from the peripheral blood of advanced HNSCC based on the cell surface expression of EpCAM; here, CTCs were detected in 27% of evaluable patients but had no association with PFS or overall survival (OS) [63]. A possible explanation for this discrepancy is the use of EpCAM as the enrichment marker, as HPV-associated cancers can harbor more mesenchymal phenotypes [3]. In another study, detection of HPV-16 E6 and E7 mRNA transcripts in CTCs also selected based on EpCAM expression was significantly associated with disease progression and mortality in a cohort of 22 patients with locally advanced OPSCC treated with CRT [64]. Studies measuring PD-L1 expression in EpCAM-selected CTCs from locally advanced HPV-driven HNSCC receiving curative-intent CRT have also been done, and results indicated that over-expression of PD-L1 in CTCs at the end of treatment was associated with worse PFS and OS [65]. Similar results have been shown in non-small cell lung cancer and metastatic genitourinary cancer patients treated with immune checkpoint inhibitors, and these data highlight a role for PD-L1 expression in mediating immune escape [66, 67].

## PERIPHERAL IMMUNE CORRELATES: DIRECT REFLECTION OF ANTI-TUMOR ACTIVITY AND INDIRECT PROXIES OF TUMOR BURDEN

### Circulating HPV-specific antibodies

Studies on the clinical utility of HPV-specific antibodies in the peripheral blood of patients with HPV-associated malignancies seem to have fallen out of favor in recent years. It is well established that antibodies against HPV-16 E6 proteins can be found in the blood sometimes 10 years before diagnosis of an HPV-driven HNSCC [68, 69], serving as an indirect marker of the presence of tumor. The sensitivity of serological assays measuring serum antibodies to early HPV-16 proteins including E6 and E7 in identifying HPV-associated cancers is on par with the sensitivity estimates of ctHPV-DNA, ranging from 83% to 96%, with high specificity as well [70–73].

Data on associations between baseline seropositivity against HPV viral antigens and recurrence are somewhat conflicted. Some studies have reported no relationship between pre-treatment anti-E6/E7 antibodies with risk of recurrence [72, 74, 75]. Conversely, in a cohort of newly diagnosed and previously untreated OPSCC treated with CRT, seropositivity for HPV proteins was strongly associated with PFS and OS [76]. A similar finding was also seen in another cohort of OPSCC, where detectable pre-treatment HPV-16 E6-specific antibodies associated with considerably reduced risk of local recurrence [77]. Data on antibody responses against other HPV viral proteins are also conflicting. One study reports a strong association between HPV-16 L1 seropositivity and better OS and PFS, in contrast to a prior report that found no relationship between anti-L1 antibody and survival [76, 78]. Of note, all these data were collected in patients with HNSCC/OPSCC receiving CRT, so the prognostic value of HPV seropositivity in other HPV-associated malignancies remains unknown.

Changes in HPV seropositivity and kinetics of HPV-specific antibodies in the setting of CRT for HPV-associated malignancies have been reported in a few studies. In particular, the association of post-treatment antibody levels with disease recurrence has been examined, but again with conflicting results. In one cohort of HPV-16+ OPSCC, circulating antibodies against HPV-16 E6 remained stable during the duration of surveillance [77], but in other cohorts of this same malignancy, antibody titers against HPV-16 and E7 decreased significantly over time in all evaluated patients [72, 74]. Seropositivity and changes in HPV-specific antibody levels were also not associated with recurrence in these studies with one exception, where patients who had disease recurrence had significantly higher antibody titers than patients with non-recurrent disease after controlling for tumor stage, though all patients did experience declines in HPV-16 E6 and E7 antibody levels over time [74]. Conversely, in another cohort of HPV+ HNSCC, patients with increasing anti-E7 antibody levels were more likely to experience disease recurrence/progression [75]. There are likely considerable differences in assay sensitivities across these studies, as they employed different systems for expressing HPV E6 and E7 proteins and serological detection. Additionally, the duration of surveillance was different across all these cohorts and the rates of disease recurrence were low, all of which together may be contributing to the discordance in results. Determining the epitopes against which HPV-specific antibodies are directed, including antibody isotype, may also help resolve the role of these antibodies in the anti-tumor response, including whether they are possibly mediating antibody dependent cellular cytotoxicity.

With rapidly evolving and combinatorial immunotherapeutic strategies for HPV-associated malignancies, it may be worth revisiting circulating HPV-specific antibodies as a peripheral biomarker of both tumor burden and generation of anti-tumor responses. Data from a cohort of HPV+ OPSCC show that titers of IgG antibodies against HPV E2, E7, and E6 proteins correlate with frequencies of HPV-specific antibody-secreting B cells in primary tumors and metastatic lymph nodes [79]. In depth serum proteomic analyses in 6 patients with HPV-associated malignancies demonstrated that patients who benefitted from treatment (CR, PR, or SD) with PD-1 immune checkpoint blockade had significantly greater serum IgG antibody levels than patients with PD, though data specific to HPV viral proteins are unavailable [80]. With the translation of HPV-targeted vaccines into clinical settings, it is prudent to evaluate circulating HPV-antibodies, as the trajectories of HPV seropositivity during immunotherapy could reflect both changes in tumor burden and development of anti-tumor responses.

### Peripheral HPV-specific T cell responses

Several studies have evaluated the development of HPV-specific T cell responses in the periphery of patients with HPV-associated malignancies treated with investigational immunotherapies, and these studies further understanding of tumor-directed activity. In one study, peripheral immune entities were associated with clinical outcomes in HPV-associated malignancies receiving immunotherapy, with a goal of identifying prognostic and predictive markers to refine patient selection and improve treatment regimens. In a cohort of patients with HPV+ malignancies treated with the experimental agent bintrafusp alfa, a bifunctional agent inhibiting PD-L1 and TGFβ, induction of HPV-16 specific CD8+ T cells after therapy was associated with clinical benefit [81]. In addition, higher baseline levels of immunostimulatory versus inhibitory plasma factors and the ratio of CD8+ T cell to myeloid-derived suppressor cell (MDSC) was positively associated with patient outcome, while increases after therapy in circulating IL-8 and the neutrophil to lymphocyte ratio (NLR) were negatively associated with outcome. Several studies have evaluated the effects of vaccines encoding HPV antigens for the treatment of HPV-associated malignancies with varying clinical results. A long peptide vaccine against HPV-16 E6/E7 targets led to CR in almost half of patients with vulvar intraepithelial neoplasms, and those patients’ developing CR had stronger IFN-producing CD4+ and CD8+ HPV-specific T cell responses than the patients who did not achieve CR [82, 83]. In a clinical investigation of a synthetic, plasmid-based DNA vaccine against HPV-16 and HPV-18 E6/E7 in cervical intraepithelial neoplasms, the authors found that 2 weeks after the third dose of this immunotherapy, the frequency of HPV-antigen specific CD137+ CD8+ T cells was associated with clinical benefit and histopathological regression of tumor [84].

Conversely, in a phase I dose escalation of a peptide vaccine against HPV-16 in recurrent or metastatic HNSCC, while half of patients developed both HPV-antigen specific T cells and HPV-specific antibody responses, no responses by RECIST were seen [85]. Similarly, in a cohort of HPV-16+ cervical cancers treated with an experimental peptide vaccine encoding HPV-16 E6/E7 proteins, broad HPV-specific and cytokine-producing T cell responses were documented but did not elicit clinical benefit [86]. A more recent study evaluating a DNA vaccine against HPV-16 and HPV-18 E6/E7 in combination with recombinant IL-12 demonstrated robust and durable antibody generation against these viral antigens as well as induction of HPV-specific cytokine-secreting and cytotoxic CD8+ T cells [87]. Notably, one patient in this trial treated with the DNA vaccine developed recurrent and metastatic disease and was then treated with nivolumab, after which the patient developed a rapid complete radiographic response. Together, these data on HPV-specific T cell responses in HPV-associated malignancies suggest that DNA and peptide vaccines encoding HPV targets readily induce HPV-specific T cell responses, but the associations of these developed immune responses with clinical outcome is not clear, largely in part due to lack of information about tumor burden at more regular intervals. Given these conflicting but sometimes very promising clinical results and the consistent development of HPV-specific T cell responses to immunotherapy, tumor burden evaluation by methods such as interrogating ctHPV-DNA in conjunction with the development of T cell responses could predict anti-tumor activity, or instead be indicative of more general immune function.

## CONCLUDING REMARKS

Liquid biopsy holds enormous potential as a surrogate of tumor burden, particularly in the setting of solid tumors where the primary tumor site is relatively inaccessible and difficult to assess frequently. In the setting of HPV-associated malignancies, information on tumor kinetics gleaned from liquid biopsies, especially during treatment with various immunotherapeutic modalities, is urgently needed to understand how and when these agents exert potential anti-tumor effects. At the present, ctHPV-DNA seems best poised for clinical utility; however, there are several technical and practical considerations that must be addressed before translation of ctHPV-DNA into a clinical setting.

The performance of ctHPV-DNA assessment versus established tissue-based methods for determining molecular HPV status is warranted. Head-to-head comparisons of primer efficiencies and studies targeting E6 and E7 proteins alone and together in a parallel or multiplexed fashion are needed for better understanding of whether evaluating multiple targets for a given HPV subtype improves assay sensitivity and specificity. There is an immediate need for data collection on the utility of ctHPV-DNA for tumor burden monitoring in immunotherapy trials, including in the settings of immune checkpoint blockade, cancer vaccines, immunocytokines, and adoptive cell therapy trials, alone and in combination to understand additive and amplified effects on tumor kinetics. Ongoing data collection of ctHPV-DNA during and after the CRT setting is warranted as well, as signs of disease recurrence and progression via ctHPV-DNA signals may necessitate additional clinical investigation and treatment. A great deal more data on the correlation between tumor size and levels of ctHPV-DNA in the periphery are also needed, including on how different CRT and immunotherapy regimens may affect the relationship between ctHPV-DNA release into the periphery and actual tumor size.

Only HPV-specific targets have been discussed at length here, as components from the HPV viral genome are ideal targets to track in this disease setting. However, incorporation of additional targets outside of the HPV genome, including *PIK3CA* and *RAS*, among other frequently mutated targets that result from HPV integration into the genome, will perhaps shed light on clinical implications of these mutations in HPV-positive tumors and to capture resistance mechanisms. Additionally, using ctHPV-DNA in the minimal/measurable residual disease setting after curative-intent treatment could increase lead time to detection of recurrent disease should it occur, allowing for a window to possibly treat the tumor without overt clinical manifestation of disease, and improve outcomes.

Combining different assays that measure peripheral surrogates of HPV-associated tumor burden is also worth exploring. Using different assays together, i.e., ctHPV-DNA plus their methylation signatures could improve tumor burden detection and reveal epigenetic signatures associated with anti-tumor responses. Combining longitudinal ctHPV-DNA with assays measuring miRNA could also do the same, and adding in CTC detection would have the added benefit of shedding light on metastases that may occur during or after CRT and immunotherapy. There may also be great value in pairing data on HPV-specific antibodies and maintenance of HPV-specific T cell responses with molecular signatures derived from ctHPV-DNA, even if these insights are not used for therapy decisions. Integrating both immune and tumor signatures in the periphery of patients with HPV-associated malignancies treated with immunotherapy could reveal the interplay between immune activity against the tumor and the tumor itself, helping in the understanding of the mechanisms of action of agents used. Such data on both real-time tumor and immune kinetics during immunotherapy could certainly be useful in guiding combinatorial approaches and developing appropriate expectations on the timeline for immunotherapy-based anti-tumor effects.

Finally, there is also a need for data in HPV-associated cancers outside of HNSCC. Most data on peripheral surrogates of tumor burden from liquid biopsies come from the HNSCC/OPSCC setting, and more extensive data are also necessary from cancers in other anatomic sites caused by high-risk HPV subtypes. It is very feasible that tumor kinetics of HPV-associated malignancies could vary depending on the site of the primary tumor, and whether patients are treated with CRT and/or an immunotherapeutic agent.

In closing, measuring the peripheral surrogates of tumor burden described here in HPV-associated malignancies with an emphasis in the immunotherapy setting is crucial for understanding clinical outcomes in this rapidly evolving field. These peripherally derived tumor signatures will allow for more frequent assessment of tumor burden during treatment, thereby affording greater opportunities to tailor treatment depending on anti-tumor activity to maximize patient benefit.
